# Effects of vagal nerve stimulation on pain frequency and intensity in chronic migraine in adults: A randomized controlled trial

**DOI:** 10.14814/phy2.70843

**Published:** 2026-04-09

**Authors:** Rabia Nasir, Mirza Obaid Baig, Turki Abualait, Sumaiyah Obaid, Irij Javed Jadoon, Fawaz Al‐hussain, Shahid Bashir

**Affiliations:** ^1^ Faculty of Rehabilitation & Allied Health Sciences Riphah International University Islamabad Pakistan; ^2^ University Institute of Physical Therapy University of Lahore Sargodha Pakistan; ^3^ College of Applied Medical Sciences Imam Abdulrahman Bin Faisal University Dammam Saudi Arabia; ^4^ Women Institute of Rehabilitation Sciences Abbottabad Pakistan; ^5^ Department Neurology College of Medicine, King Saud University Riyadh Saudi Arabia; ^6^ Research Center, King Fahad Specialist Hospital Dammam Dammam Saudi Arabia

**Keywords:** chronic pain, headaches, migraine, vagal nerve stimulation

## Abstract

To determine the effects of noninvasive vagal nerve stimulation (nVNS) on the frequency, intensity, impact, and quality of life in individuals with chronic migraine. The randomized controlled trial (RCT) was conducted over 128 participants, randomly assigned to either the experimental or control group. The experimental group received nVNS, while the control group underwent sham interventions along with the prescribed medications. Data collection involved the pre and post‐use of a structured headache diary, a numerical pain scale (NPS), the multidimensional pain inventory, and migraine‐specific quality of life (MS‐QoL) questionnaires. The experimental group's MS‐QoL scores significantly improved, with a mean rank of 94.59, and *p* value <0.001. Similarly, the NPS scores showed that the experimental group's pain levels were considerably lower, with a mean rank of 36.77 and *p* value <0.001. The MPI scores highlighted a substantial reduction in pain‐related interference and behaviors within the experimental group, with *p* value = 0.035. In terms of headache severity, the structured headache diary revealed that the experimental group experienced fewer severe headaches, with only 16 instances (15.6%), and an increase in moderate headaches to 51 instances (51.6%). The study indicates that nVNS significantly enhanced quality of life and reduced pain intensity, frequency, and impact among chronic migraine patients over 4 weeks of active stimulation.

## 1 INTRODUCTION

Migraine, a prevalent and debilitating brain disorder, presents in various forms, primarily categorized into episodic migraine (EM) and chronic migraine (CM). EM is defined by experiencing fewer than 14 headache days per month, whereas CM involves 15 or more headache days per month over 3 months, with symptoms characteristic of migraines. Understanding the epidemiology, pathophysiology, and potential treatments for both forms is essential for improving patient outcomes and addressing the substantial public health burden associated with migraines (Aurora & Brin, 2017; Chalmer et al., 2020).

The global prevalence of migraines is significant, affecting around 15% of the population annually. However, the prevalence varies by region, with the highest rates observed in Southeast Asia, ranging from 25% to 35%. These statistics underscore the need for effective management strategies and treatments to mitigate the impact of migraines on individuals and healthcare systems. The overall costs associated with CM are roughly four times higher than those of EM, emphasizing the need for effective treatment and management strategies (Ashina et al., 2021; Burch et al., 2021; Nesbitt & Goadsby, 2012). Neuromodulation has emerged as a promising area of research, particularly noninvasive vagus nerve stimulation (nVNS), which offers potential relief without the complications associated with invasive procedures (Ashina et al., 2021; Nesbitt & Goadsby, 2012).

The pathophysiology of migraines involves complex brain mechanisms. The brainstem plays a crucial role in regulating pain signals within the trigeminocervical complex. Chronic migraine can develop due to activity‐independent sensitization of pain pathways, resulting from repeated migraine attacks. Medication overuse is a common issue among migraine sufferers, leading to headaches resembling chronic migraine through adaptive changes in the brain. This highlights the need for alternative treatment approaches, such as neuromodulation. Vagus nerve stimulation (VNS), particularly noninvasive forms like nVNS, has shown promise in modulating neurotransmitter levels, cerebral metabolism, and blood flow, contributing to pain relief in various pain models (Burch et al., 2021; Martelletti et al., 2013).

The cervical nVNS device generates a low‐voltage electrical signal, delivered to the neck area near the vagus nerve, while transcutaneous auricular VNS devices target the outer ear's concha, providing a non‐invasive treatment option for patients (May & Schulte, 2016; Munakata et al., 2009).

The vagus nerve plays a crucial role in regulating functions like breathing, heart rate, and digestion. Comprising 20% efferent fibers and 80% afferent fibers, it transmits sensory information from various body parts to the brain. Existing cadaveric work, though limited, indicates that ABVN fibers predominantly supply the concha and inner tragus; other ear regions receive mixed innervation from non‐vagal nerves. The auricular branch of vagal nerve is primarily afferent, with myelinated and unmyelinated fibers comparable to the rest of the vagus, supporting transmission of stimulation from skin to brainstem (Butt et al., 2020). Vagus nerve stimulation is believed to modulate nociceptive transmission by activating afferent fibers that relay sensory input to brain regions involved in pain processing. These include the nucleus tractus solitarius, area postrema, spinal trigeminal nucleus, locus coeruleus, periaqueductal gray, raphe magnus, thalamus, and hypothalamus (Straube et al., 2015).

Research has shown that VNS can influence neurotransmitters, regulate cerebral metabolism, and modulate pain pathways, contributing to its analgesic effects. Additionally, tVNS has been utilized in treating severe cases of epilepsy and depression, demonstrating its potential benefits in managing chronic pain conditions (Tassorelli et al., 2018).

nVNS has emerged as a promising non‐pharmacological option for migraine, but the evidence base remains mixed: a comprehensive 2020 systematic review and meta‐analysis (Moisset et al., 2020) concluded that VNS‐based neuromodulation showed no significant preventive effect with high heterogeneity across trials, limiting firm conclusions about prophylaxis, whereas a 2023 meta‐analysis (Song et al., 2023) reported more consistent benefits for acute treatment and only modest, statistically inconsistent effects for prevention; notably, low‐frequency auricular nVNS appeared to reduce monthly migraine days and headache intensity in some analyses, yet results varied by device, parameters, and study design. Methodological challenges that likely contribute to these discrepancies include small samples, short follow‐up windows, difficulties in designing credible shams/blinding, and protocol heterogeneity in stimulation frequency, pulse width, dosing, and adherence monitoring. At the same time, recent syntheses characterize nVNS, particularly auricular approaches, as safe and well tolerated, with adverse effects typically mild and transient (for example, local tingling or minor skin irritation), supporting further controlled evaluation. Against this backdrop, the present trial is designed to address a specific gap in the literature by providing supervised, clinic‐delivered, fully adherent testing of a standardized, low‐frequency auricular stimulation protocol over a defined short‐term horizon, with transparent reporting of effect sizes and prespecified outcomes; by minimizing noncompliance and procedural variability, two sources of bias frequently cited in prior reviews, this study contributes high‐fidelity data to clarify the short‐term clinical impact of auricular nVNS while acknowledging that longer follow‐up will be needed to determine durability.

## 2 METHODS

### 2.1 Participants

The Randomized Controlled Trial (RCT), including both experimental and control groups, was conducted at Mubarak Medical Complex, Sargodha, from December 2023 to August 2024 after the ethical approval of Research Ethical Committee, Riphah International University (Riphah/RCRAHS‐ISB/REC/MS‐PT/01746) registered with clinicaltrial.org (NCT06191016). The experiment was conducted in accordance with the Helsinki Declaration. After obtaining the written informed consent from the participants, the data on demographics and relevant information was taken. The inclusion criteria included the person of either gender with more than 18 years of age having chronic migraine according to the (International Classification of Headache Disorder, edition 3 (ICHD‐3)) (International Headache Society, 2013), (headache for 15 or more days per month over a span exceeding 3 months) with/without aura. The exclusion criteria include the person with mental illness, sensitivity to light and brain tumors.

### 2.2 Sample size

**FIGURE 1:**
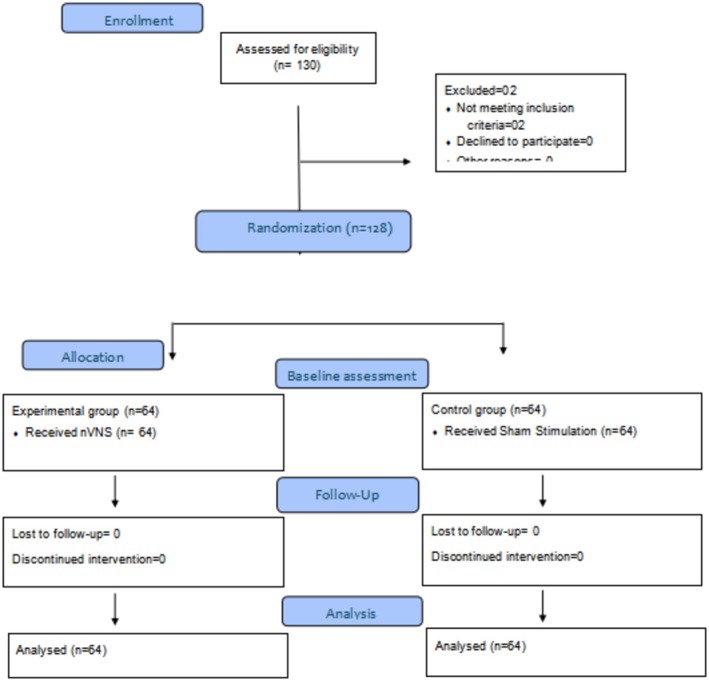
CONSORT diagram.

A sample size of 128 participants was determined using G*Power software, with an effect value of 0.5, an alpha error of 0.05, and a statistical power of 0.80. A total of 130 participants were initially assessed, with two excluded for not meeting the chronic migraine criteria, resulting in 128 participants being randomized into the two groups (Figure 1).

### 2.3 Randomization

We employed sequentially numbered, opaque, sealed envelopes (SNOSE) that were prepared by an independent administrator who was not otherwise involved in the study to guarantee robust allocation concealment and prevent selection bias. Envelopes were opaque, tamper‐evident, and opened only after participant enrollment in accordance with a chronological sequence. This method guaranteed that investigators who were responsible for recruitment or baseline assessment could not anticipate group assignment.

Despite participants not being completely blinded due to the sensory aspect of active stimulation, outcome assessors remained unaware of group assignments throughout the trial. The staff who gave out the questionnaires were not the same people who ran the stimulation sessions, and they were told not to ask about or talk about how therapy was assigned. Data entry workers and analysts were similarly kept in the dark until the database was locked.

### 2.4 Intervention

Participants in the experimental group received the tVNS by using the Premiere Combo Plus, Everyday Medical Instruments Company, Taiwan, through the tragus, an external part of the ear, via the clip electrode at a 200‐millisecond pulse width in normal mode, operating at 30 Hz. The intensity was adjusted for patient comfort, with two sessions of 2 min each, spaced 5 to 10 min apart (Yap et al., 2020). Stimulation sessions included 2‐min intervals with 2–3‐min rest periods, followed by another 2‐min stimulation cycle, conducted over 4 weeks.

All stimulation sessions were conducted on‐site at the hospital under direct supervision by trained research staff. Participants attended the facility for every scheduled treatment session. Because stimulation was delivered in person, research personnel ensured correct electrode placement, appropriate device parameter settings, and adherence to the full prescribed session duration.

The device does not incorporate internal data‐logging capabilities; therefore, automated extraction of session‐level usage metrics was not feasible. However, supervised administration allowed for complete verification of attendance and adherence, and no sessions were missed during the study period. Staff recorded session completion in standardized logs immediately following each supervised treatment to ensure fidelity to the intervention protocol. This supervised approach minimized the risk of noncompliance, incorrect device operation, or deviation from the prescribed intervention schedule.

The sham protocol was engineered to be physiologically inactive by stimulating the earlobe, an area not innervated by the auricular branch of the vagus nerve. Only some auricular areas, namely the tragus and cymba conchae, possess vagal afferent fibers that may convey vagal impulses. Recent systematic evaluations indicate that stimulation beyond ABVN regions does not engage vagal pathways, making it suitable as a sham control in taVNS research (Moisset et al., 2020).

### 2.5 Outcome tools

The outcome tools included the numeric pain scale (NPS) for pain, multidimensional pain inventory (MPT) for pain‐related behaviors as primary outcome, and migraine‐specific quality of life (MSQ) version 2.1 were considered as secondary outcome and used as pre and post intervention. Additionally, the paper‐based structured headache diary was also implemented 1 month prior to the intervention and at the 4th week of intervention, to observe the features, number of episodes, severity and triggering/relieving factors of migraine.

### 2.6 Statistical analysis

The data analysis was made through SPSS version 25. All participants completed the full intervention protocol with no dropouts or missing outcome data; therefore, all analyses were performed on the complete dataset. Although no formal intention‐to‐treat procedures (such as imputation) were necessary, the analysis effectively represents an intention‐to‐treat population because all enrolled participants were included in all analyses. The pain and quality of life were found to be not normal, hence we opted for the Mann–Whitney *U* test and Wilcoxen Sign Ranked test for between‐group and within the group analysis, respectively. While the pain‐related behaviors were found to be normally distributed hence analyzed via independent and paired *t*‐test for between‐group and within‐group analysis.

## 3 RESULTS

The baseline characteristics of participants in the experimental and control groups are shown in Table 1. The mean age was 26.84 ± 6.22 years in the experimental group and 27.32 ± 6.35 years in the control group. There were more females with 53.1% and 59.4% in the control and experimental groups, respectively (Table 1).

**TABLE 1:** Baseline characteristics of participants.

Variables	Groups		
	Experimental group	Control group	*p* Value
	Mean ± SD	Mean ± SD	
Age (Years)	26.8438 ± 6.221	27.3281 ± 6.354	0.385

		*n*	Percent %	*n*	Percent %	
Gender	Male	30	46.9%	26	40.6%	0.476
	Female	34	53.1%	38	59.4%	
Occupation	Student	10	15.6%	13	20.3%	0.693
	Teacher	10	15.6%	12	18.8%	
	Physiotherapist	19	29.7%	20	31.3%	
	Worker	14	21.9%	8	12.5%	
	Housewife	11	17.2%	11	17.2%	
Marital status	Single	26	40.6%	26	40.6%	1.000
	Married	38	59.4%	38	59.4%	
Educational status	Intermediate	10	15.6%	13	20.3%	0.703
	Undergraduate	40	62.5%	43	67.18%	
	Masters	14	21.9%	8	12.5%	
Employment status	Unemployed	28	43.8%	31	48.4%	0.595
	Employed	36	56.3%	33	51.6%	
Site of involvement	Right temporal	10	15.6%	13	20.3%	0.662
	Frontal area	10	15.6%	12	18.8%	
	Right temporal and periorbital	19	29.7%	18	28.1%	
	Orbital	14	21.9%	8	12.5%	
	Left temporal	11	17.2%	13	20.3%	
Migraine duration	10–20 h	10	15.6%	13	20.3%	0.703
	20–30 h	11	17.2%	12	18.8%	
	30–40 h	18	28.1%	20	31.3%	
	40–50 h	14	21.9%	8	12.5%	
	51 and above hours	11	17.2%	11	17.2%	
Dominant side	Right‐handed	38	59.4%	40	62.5%	0.717
	Left‐handed	26	40.6%	24	37.5%	

At baseline, there was no significant difference in MSQ scores between the experimental and control groups (*p* = 0.823). By Week 4, the experimental group had a markedly elevated mean rank in comparison to the control group (94.59 vs. 34.41; *p* < 0.001), signifying superior functional enhancement. The effect size (*r* = 0.95) shows a very large shift, which shows that the difference is clinically important, not just statistically significant. Likewise, there was no substantial group difference at baseline (*p* = 0.247). The experimental group had far lower pain scores than the control group after 4 weeks, as seen by a lower mean rank (36.77 vs. 92.23; *p* < 0.001). The comparable effect size (*r* = 0.98) once more indicates a substantial effect, illustrating a significant treatment‐related alleviation of pain symptoms (Table 2).

**TABLE 2:** Between‐groups analysis.

Outcome	Time point	Experimental group		Control group		*p* Value	Effect size (*r*)
		Mean rank	Sum of rank	Mean rank	Sum of rank		
MSQ	Baseline	68.65	4393.50	60.35	3862.50	0.823	0.95
	4 weeks	94.59	6053.50	34.41	2202.50	**<0.001**	
NPS	Baseline	65.95	4220.50	63.05	4035.50	0.247	0.98
	4 weeks	36.77	2353.50	92.23	5902.50	**<0.001**	

*Note*: Bold values indicate the level of significance.

At baseline, no significant difference existed between the experimental and control groups (142.71 ± 21.47 vs. 149.82 ± 14.63; *p* = 0.562), with a minimal effect size (Cohen's d = 0.095), suggesting near equality between the groups prior to the intervention. After 4 weeks, both groups exhibited comparable mean MPI scores (138.40 ± 23.49 vs. 140.52 ± 20.99), with a statistically significant difference (*p* = 0.035). Nonetheless, as *p* values solely indicate the presence of a difference rather than its magnitude, interpretation must depend on effect size. The Cohen's d for this comparison, derived from the reported means and standard deviations, suggests that the effect size is tiny, indicating that the statistical significance does not reflect a substantial or clinically meaningful improvement. Consequently, although a quantifiable difference was seen, the impact size indicates that the shift is minor and warrants careful interpretation (Table 3).

**TABLE 3:** Between‐group analysis.

Variable	Time frame	Groups	Mean ± SD	*p* Value	Cohen's *d*
Multidimensional Pain Inventory (MPI)	Baseline	Experimental	142.71 ± 21.47	0.562	0.095
		Control	149.82 ± 14.63		
	Week 4	Experimental	138.40 ± 23.49	**0.035**	
		Control	140.52 ± 20.99		

*Note*: Bold values indicate the level of significance.

In the experimental group, MSQ improved from baseline to Week 4 (*Z* = 6.96, *p* < 0.001, *r* = 0.87) and NPS decreased over the same interval (*Z* = 6.96, *p* < 0.001, *r* = 0.87). In the control group, pre‐post change was not significant for MSQ (*Z* ≈0.64, *p* = 0.520, *r* ≈0.08) or for NPS (*Z* ≈1.16, *p* = 0.247, *r* ≈0.15) (Table 4).

**TABLE 4:** Within‐groups analysis.

Outcome	Time point	Experimental group (*N* = 64)					Control group (N = 64)				
		Mean rank	Sum of rank	*Z*	*p* Value	Effect size (*r*)	Mean rank	Sum of rank	*Z*	*p* Value	Effect size (*r*)
MSQ	Baseline	32.5	2080	6.96	**<0.001**	0.87	62.5	4000	0.64	0.520	0.08
	4 weeks	28.9	2567				61.0	3423			
NPS	Baseline	20.4	2156	6.96	**<0.001**	0.87	65.9	5390	1.16	0.247	0.15
	4 weeks	16.5	1056				64.5	4128			

*Note*: Bold values indicate the level of significance.

In the experimental group, MPI decreased from 141.48 ± 14.98 at baseline to 137.83 ± 20.99 at Week 4 (*p* < 0.001), yielding a small standardized mean change *d*_av_ = −0.20. In the control group, MPI changed from 146.35 ± 12.76 to 145.88 ± 19.54 (*p* = 0.142), corresponding to a negligible *d*_av_ = −0.03. Because *p* values indicate statistical evidence but not magnitude, we emphasize the effect sizes and provide descriptive summaries at both time points for clinical interpretability (Table 5).

**TABLE 5:** Within the group analysis.

Variable	Groups	Time frame	Mean ± SD	*p* Value	Effect size $d_{\mathrm{av}}$
Multidimensional pain inventory (MPI)	Experimental group	Baseline	141.4844 ± 14.98	**<0.001**	**0.203**
		Week 4	137.8281 ± 20.99		
	Control group	Baseline	146.345 ± 12.76	0.142	0.029
		Week 4	145.875 ± 19.54		

*Note*: Bold values indicate the level of significance.

There were 50% of migraine episodes turned into the mild category after 4 weeks of intervention in the experimental group in contrast of 6.2% in control group. There was a marked reduction in moderate and severe cases while there was an increase in cases of severity in control groups after 4 weeks (Table 6).

**TABLE 6:** Migraine severity; influence of intervention.

	Experimental group		Control group	
	Baseline	Week 4	Baseline	Week 4
Mild (*n*/%)	0/0	32/50	0/0	4/6.2
Moderate (*n*/%)	28/42.9	19/29.6	35/54.6	28/43.7
Severe (*n*/%)	36/57.1	13/20.3	29/45.3	32/50

Table 7 displays the frequency and proportion of reported triggering and alleviating factors at baseline and Week 4 for both the experimental and control groups. Values are presented as *n* (%), with percentages rounded to the nearest integer for clarity and uniformity. All percentages were computed based on the total number of participants in each group (*N* = 64). Due to rounding of percentages, the totals may not accurately sum to 100%. The table facilitates a direct comparison of temporal changes both within and between groups for each variable.

**TABLE 7:** Triggering and relieving factors at baseline and week 4 in experimental and control group based on *N* = 64 per group.

	Experimental group		Control group		*p* Value
	Baseline	Week 4	Baseline	Week 4	
Triggering factors *n* (%)					
Fatigue	7 (11)	5 (8)	3 (5)	4 (6)	0.514
Exercise	5 (8)	10 (16)	5 (8)	7 (11)	0.655
Heat	7 (11)	12 (19)	10 (16)	8 (13)	0.253
Stress	22 (34)	18 (28)	12 (19)	13 (20)	0.582
Medication (missed)	8 (13)	11 (17)	9 (14)	12 (15)	0.961
Meal (missed)	9 (14)	0 (0)	15 (23)	12 (19)	0.051
Salt	5 (8)	5 (8)	5 (8)	6 (9)	0.834
Caffeine	0 (0)	2 (3)	3 (5)	4 (6)	0.778
Nuts	0 (0)	0 (0)	1 (2)	2 (3)	0.709
Chocolate	1 (2)	1 (2)	1 (2)	0 (0)	1.0
Relieving factors (*n*/%)					
Rest	18 (28)	13 (20)	12 (19)	9 (14)	0.947
Darkroom	5 (8)	16 (25)	15 (23)	25 (39)	0.279
Medications	4 (6)	3 (5)	7 (11)	8 (13)	0.647

*Note*: Percentages rounded to the nearest whole number; totals may not add to 100% due to rounding.

## 4 DISCUSSION

The objective of the study was to evaluate the effectiveness of noninvasive vagus nerve stimulation (nVNS) in chronic migraines. The results demonstrated significant improvements in the experimental group that received nVNS, highlighting its potential as a viable treatment for chronic migraine sufferers.

Real‐world evidence provides support for the effectiveness of nVNS as an acute and preventive treatment for cluster headache. In a retrospective analysis of data from 30 patients with cluster headache, nVNS led to significant decreases from baseline in mean attack frequency (64%), duration (43%), and severity (23%) (Moisset et al., 2020). The efficacy of nVNS for acute migraine treatment was assessed in an open‐label 6‐week pilot study (Goadsby et al., 2014). Among the 19 patients who had moderate or severe headaches at baseline for their first attack, four (21%) were pain‐free, and nine (47%) reported pain relief 2 h after nVNS monotherapy. Similar results were observed in the current study may be due to the similar population.

In a review concerning neuromodulation techniques for acute and preventive migraine treatment, the authors concluded that vagus nerve stimulation had no significant effect and the heterogeneity was high (Lai et al., 2020). In another review, analyzed 6 published randomized controlled trials on the effect of nVNS on migraine and cluster headache. They stated that there is a significant effect of nVNS on treatment of acute migraine and cluster attacks, but no significant effect on headache days in episodic migraine (Silberstein et al., 2020). This is might be due to the differences in the characteristics of the participants including the migraine type, involvement of aura, and the dosages of the nVNS. Additionally, the vagal stimulation should be seen as a preferred option in the treatment of acute migraine attacks or cluster attacks in episodic cluster headache, as well as an option in the preventive treatment of chronic cluster headache (Silberstein et al., 2020). There was no difference in pain intensity, the number of migraine attacks, and frequency after the stimulation using at‐VNS with 1 Hz (Cao et al., 2021; Luo et al., 2020; Sacca et al., 2023; Zhang et al., 2019, 2021). The reports contrast with the current study may be due to different outcome tools, varied treatment doses, and especially the frequency of stimulation. nVNS can increase the connectivity between the motor‐related thalamus subregion and anterior cingulate cortex/medial prefrontal cortex, and decrease the connectivity between the occipital cortex‐related thalamus subregion and postcentral gyrus/precuneus, resulting in easing the symptoms of headache/migraine (Sacca et al., 2023). VNS modulates activity in different brain regions through pathways such as the nucleus solitarius and locus coeruleus, thereby regulating migraine‐related symptoms (Zhang et al., 2021). In addition, the brainstem trigeminal nervous system directly or indirectly associated with the vagus nervous system, is involved in migraine, and VNS may modulate pain by inhibiting trigeminal nervous system discharge (Song et al., 2023). nVNS may affect trigeminal nerve activity by modulating excitatory and inhibitory neurotransmitters, but may also be associated with peripheral sensitization‐related proteins, such as P‐ERK in the trigeminal nerve (Frangos & Komisaruk, 2017). These underlying mechanisms provide theoretical support for the n‐VNS treatment of migraine and might be the reason for the influential results of the experiment.

While numerous preliminary open‐label and pilot trials indicate possible advantages of noninvasive vagus nerve stimulation for migraine, bigger systematic reviews present more ambiguous findings. Moisset et al. (2020) discovered no significant preventative benefit of vagus nerve‐based neuromodulation and observed considerable heterogeneity across the trials examined. Likewise, Song et al. (2023) determined that nVNS exhibited a more pronounced advantage for acute treatment (e.g., enhanced ≥50% responder rate) but revealed only modest and statistically inconsistent outcomes in migraine prevention, with no significant decrease in monthly migraine or headache days for cervical nVNS. Consequently, our findings must be contextualized within the wider body of evidence, recognizing that although nVNS has promise, particularly for acute treatment, its preventive effectiveness in chronic migraine is not yet adequately substantiated.

Although the results are encouraging statistically, there are certain limitations of the study, including the single data collection site, which may cause demographic discrepancies and affect the results. Additionally, the methods did not consider the objective data, including the electrophysiological parameters, hence it is difficult to state about the brain state/changes during and after the stimulation. The outcomes relied primarily on subjective, patient‐reported measures, which may be influenced by individual perception, recall bias, or expectancy effects. However, the study showed good retention of the participants, resulting in no dropouts during the intervention time. The relatively brief follow‐up period in this trial limits the interpretation of longer‐term effects of nVNS on migraine outcomes. This is particularly relevant given that existing systematic reviews of nVNS for migraine describe considerable heterogeneity and inconsistent evidence regarding preventive efficacy over extended durations. For instance, Moisset et al. (2020) reported that VNS‐based neuromodulation showed no significant long‐term preventive benefit, partly due to high heterogeneity and limited sustained‐effect data in available trials. Similarly, Song et al. (2023) found that while the acute benefits of nVNS were more consistent, the prophylactic effects remained modest and not statistically robust over longer observation periods. Accordingly, our findings should be interpreted as short‐term outcomes, and longer, adequately powered longitudinal studies are needed to clarify whether the clinical improvements observed here persist beyond the initial treatment window.

This study was conducted without participant blinding, which increases the risk of expectation‐driven bias, particularly relevant in pain‐modulation studies. The absence of blinding reflects a well‐recognized challenge in tVNS research, where even minimal electrical stimulation produces perceptible sensations, making the development of indistinguishable sham conditions difficult. The literature acknowledges that tVNS methodologies vary widely between research groups and that optimal stimulation and control parameters remain non‐standardized, further complicating the establishment of reliable sham protocols.

Lastly, the use of a commercial TENS‐based device (Premier Combo Plus) imposed technical constraints on stimulation parameters and precluded implementation of an active‐sham mode. Although stimulation settings were selected to align with ranges frequently reported in tVNS studies, the device's limitations may restrict generalizability to dedicated neuromodulation platforms. While supervised delivery reduced protocol deviations, future research with extended follow‐up, objective biomarkers, and blinded sham‐controlled designs will be crucial to definitively ascertain the enduring clinical efficacy of this intervention.

A major limitation of this study is the lack of direct physiological verification of autonomic engagement. Although VNS is commonly interpreted within a vagally mediated framework, no objective indices of autonomic function (such as heart rate variability, arterial blood pressure responses, or baroreflex sensitivity) were collected. As a result, any mechanistic interpretations regarding autonomic modulation must be considered hypothetical. Accordingly, the present findings are more appropriately interpreted within a clinical behavioral context rather than as evidence of specific neurophysiological mechanisms. This constraint limits causal inferences concerning the biological pathways underlying the observed effects.

## 5 CONCLUSION

nVNS demonstrated promising clinical benefits in the management of chronic migraine, including reduction in pain intensity, pain‐related interventions and behaviors, and improvements in quality of life. Participants receiving nVNS also showed reduced reliance on pharmacological treatment. However, in the absence of physiological measures confirming autonomic modulation, the underlying mechanisms remain uncertain. These findings support nVNS as a potentially useful nonpharmacological adjunct for long‐term migraine management, warranting further investigation in studies incorporating objective mechanistic assessment.

## AUTHOR CONTRIBUTIONS

**Rabia Nasir:** Data curation; formal analysis; methodology. **Mirza Obaid Baig:** Conceptualization; methodology; project administration; supervision. **Turki Abualait:** Formal analysis; methodology. **Sumaiyah Obaid:** Methodology. **Irij Javed Jadoon:** Formal analysis; writing – original draft; writing – review and editing. **Fawaz Al‐hussain:** Methodology. **Shahid Bashir:** Methodology; validation.

## FUNDING INFORMATION

The authors have nothing to report.

## ETHICS STATEMENT

The study was carried out in accordance with the Declaration of Helsinki. This study was with the ethical approval of Research Ethical Committee of Riphah International University (Riphah/RCRAHS‐ISB/REC/MS‐PT/01746) and registered at clinicaltrial.gov with the unique ID: NCT 06191016.

## Data Availability

The datasets used and/or analyzed during the current study are available from the corresponding author on reasonable request.
